# A Review of Nutrition, Bioactivities, and Health Benefits of Custard Apple (*Annona squamosa*): From Phytochemicals to Potential Application

**DOI:** 10.3390/foods14193413

**Published:** 2025-10-02

**Authors:** Ningli Qi, Xiao Gong, Yang Luo, Chenghan Zhang, Jingjing Chen, Tinghui Chen

**Affiliations:** 1 Sanya Research Institute, Chinese Academy of Tropical Agricultural Sciences, Sanya 572019, China; 2 Agricultural Products Processing Research Institute, Chinese Academy of Tropical Agricultural Sciences, Zhanjiang 524001, China; 3 College of Notoginseng Medicine and Pharmacy, Wenshan University, Wenshan 663099, China; 4 College of Food Science and Technology, Huazhong Agricultural University, Wuhan 430070, China

**Keywords:** *Annona squamosa*, nutritional composition, phytochemicals, health-promoting, applications progress

## Abstract

The custard apple (CA) is a noble fruit in tropical regions worldwide. It has attracted a growing interest due to its organoleptic properties and nutritional value. With the expansion of international trade, both its cultivation and consumption have grown significantly in recent years. Previous researchers have sporadically investigated its nutritional composition and health benefits; however, existing information on its processing and utilization is highly fragmented and lacks a comprehensive overview of its constituents, biological activities, and potential applications. This review is a detailed summary of the nutritional and bioactive properties, safety evaluations, and potential applications of CA. Following PRISMA guidelines, peer-reviewed studies published between 2000 and 2025 were systematically searched in PubMed, Scopus, ResearchGate, and Web of Science. Inclusion criteria comprised studies reporting on nutritional composition, phytochemicals, bioactivities, health promotion, and applications of CA. In addition to primary nutrients like carbohydrates, protein, fatty acids, vitamins, and minerals, CA also contains a multitude of bioactive compounds, mainly including phenols, flavonoids, terpenoids, acetogenins, and alkaloids, which are attributed to a range of health benefits, such as antioxidant, anti-microbial, anti-tumor, blood sugar regulation, and cognitive function improvement. However, more clinical and toxicological profiles remain underexplored, and future research should focus on standardized extraction, safety evaluation, and translational applications. Additionally, the challenges and future perspectives in industrial applications are discussed, which are expected to offer comprehensive information for the utilization of CA.

## 1. Introduction

**Figure 1:**
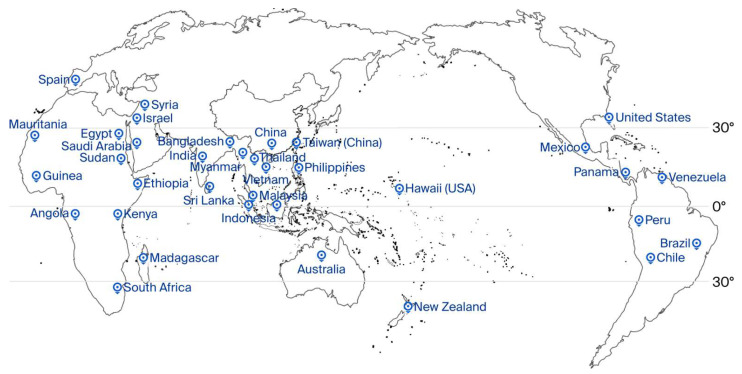
Major global producing regions of the custard apple.

*Annona*, derived from the Latin phrase ‘annual harvest’, belongs to the family Annonaceae with about 2500 species and more than 130 genera. *Annona squamosa*, considered to have originated in the New World tropics, is the most widely cultivated variety [1]. The small tropical tree was first bred in Florida in 1908, and now it has been spread worldwide. To date, five main varieties of *Annona* spp. (*A. squamosa*, *A. atemoya*, *A. cherimola*, *A. muricata*, and *A. reticulata*) have been commercially cultivated in the world. Regional names of the fruit include the custard apple (CA), sitaphal (India), anon (Portuguese), anona (Israel/Lebanon), shijia (Chinese mainland), and pineapple sugar apple (Taiwan, China) [2]. It inherits fine qualities, such as having a small quantity of seeds, easy preservation after harvest, and rare cracking, garnering widespread attention. The major global producing regions are shown in Figure 1, and India is the largest producer of CA in the world. As reported by the NHB (National Horticulture Board, India), approximately 55,000 hectares are under CA cultivation, with production of the fruit reaching 38,726,000 tons in 2023; exports of the fruit were approximately 1200 tons, earning more than 1.06 million dollars. Taiwan, China follows with approximately 5500 hectares under CA cultivation and an output value of 603 million dollars. Volza’s Global Export Data shows that 2023 exports grew 45 percent year on year, with India, Vietnam, and Colombia as top exporters. The average international market price is between 4 and 6 dollars per kilogram, peaking at 10 dollars per kilogram due to high transportation costs, making CA a high-value fruit.

This review mainly focuses on providing baseline information of the nutritional and bioactive properties, safety evaluations, and potential applications of CA (e.g., pulp, seed, and leaf). Following PRISMA guidelines, peer-reviewed studies published between 2000 and 2025 were systematically searched in PubMed, Scopus, ResearchGate, and Web of Science. Inclusion criteria comprised studies reporting on the nutritional composition, phytochemicals, bioactivities, health benefits, and applications of CA (Figure S1). The fruit is popular for its delicious aromatic white pulp and richness in vitamin C, vitamin A, vitamin B_6_, potassium, magnesium, copper, citric acid, malic acid, and dietary fibers. It is usually used in fruit cups, salads, milkshakes, and widely used in juice, beverage, and puree production, as well as in baked goods recipes like cakes and tarts and frozen desserts. Furthermore, its importance in traditional medicine and healthcare is gaining attention. Different parts of the CA (pulp, peel, seed, leaves, bark, and roots) are utilized for prevention and auxiliary treatment of diseases, attributed to bioactive ingredients such as phenolic compounds, flavonoid compounds, glycosides, terpenoids, steroids, and alkaloids [3]. Thus, CA has immense nutraceutical and therapeutic potential in plant food, especially in food for special medical purposes (FSMPs), prompting extensive global research. This review details the recent progress, discusses agri-food and pharmaceutical applications, proposes processing industry challenges and future perspectives, and aims to offer comprehensive information for product innovation and industrial structure upgrading.

## 2. Chemical Composition and Nutritional Profiles

Custard apple pulp (CAP) is sweet-flavored, juicy, and contains moisture (8.36%), proteins (1.6%), carbohydrates containing mainly soluble sugar (23.9%), soluble solids like sugar and organic acids (18.0–26%), fat acids (0.1–0.3%), and minerals (0.7%) (Table 1). Custard apple leaves (CAL) contain phenols, alkaloids, etc., demonstrating potential anti-microbial, antioxidant, and lipid-lowering properties [4]. The extract of CAP possesses significant antioxidant and anti-microbial activities, is effective against various pathogenic bacteria [5], and exhibits stronger inhibitory effects on prostate and colorectal cancer cells [6]. The extract of CAL demonstrates broad potential health benefits, including antioxidant and antiandrogenic effects, particularly showing therapeutic potential for benign prostatic hyperplasia (BPH) [7].

### 2.1. Fatty Acids Profile

Fatty acids (FAs) are vital energy-storage compounds and primary components of plant membrane lipids; their composition modulates membrane properties like fluidity. The FA composition of lipids in mesocarp tissues has a crucial role during the ripening and senescence processes of the CA fruit. Palmitic acid (C16:0), stearic acid (C18:0), oleic acid (C18:1), and linoleic acid (C18:2) were the major FAs in the edible parts; and the highest level of FAs, especially unsaturated ones, was found in custard apple seed (CAS) [19].

### 2.2. Mineral Elements

Mineral elements in fruit participate in cellular composition and metabolic regulation, such as chlorophyll biosynthesis (especially Cu and Mg) and tissue softening (mainly Ca). The CA fruit is rich in Mg (approximately 14% RDI) and K (approximately 32% RDI), exceeding those of many fruits, such as bananas, coconuts, kiwis, etc. [17]. High levels of Mg are perfect for unwinding and soothing the heart muscles, possibly improving cardiovascular health and reducing the risk of heart attacks and strokes. A well-balanced ratio of Na and K helps to promote healthy dilation of the veins and arteries, which in turn leads to better blood circulation.

### 2.3. Essential Oils

Essential oils (EOs) are aromatic, volatile, and concentrated plant extracts that retain or magnify the natural smell and flavor of their source and exhibit biological activities like antioxidative, analgesic, anti-microbial, anti-inflammatory, and anticancer ones. EOs from the CA fruit, mainly including α-pinene, 1,8-cineole, β-pinene, limonene, linalool, (E)-caryophyllen, myrcene, p-cymene, and caryophyllene oxide, have been used for the manufacture of cosmetics, functional food, and natural medicines [20].

### 2.4. Vitamins

CA is rich in vitamins, including provitamin A, B complex, C, and E. Provitamin A carotenoids supplement retinol requirements; lutein (B_9_) is most abundant in peel. CA serves as a natural pyridoxine (B_6_) source, aiding neurotransmitter production and alleviating mood symptoms from deficiency [21]. The content of α-tocopherol (V_E_) ranged from 0.07 to 0.23 mg/100 g of pulp, while values varied from 0.48 to 0.62 mg/100 g in the peel, and the highest content (1.10 mg/100 g) was reported in the seeds.

### 2.5. Carbohydrates

Carbohydrates in CAP primarily include glucose, fructose, sucrose, and oligosaccharides. The polysaccharides, mainly acidic heteropolysaccharides with high galacturonic acid, reach 4.26–13.01% (dry weight) [22]. For fruit processing, it is necessary to select these fruits with high contents of soluble solids and ascorbic acid, but for fresh consumption, juicy and big-sized fruits are commonly preferred.

### 2.6. Volatile Profiles

Fruit flavor is a key factor for consumer acceptance. Fresh fruits of CA contain 39 volatile compounds [23]. Aroma evolves with maturity, and early stages feature 1-pentanol, hexanol, trans-2-hexenol, 3-methylbutanal, and 2,3-pentanedione; during ripening, α-terpineol and linalool increase while acetone and ethanol decrease; full maturity brings ethyl acetate, ethyl butanoate, and other esters that form characteristic floral-fruity notes.

In conclusion, CAS is a potential resource of fiber, protein, and fatty acids, the values of which are approximately 2.5, 18, and 96 times of those in CAP, respectively. Moreover, CAF is also rich in dietary fiber and can serve as a low-calorie source of dietary fiber.

## 3. Phytochemical Compounds

CA fruits are excellent sources of health-promoting compounds that are active in neutralizing reactive oxygen (ROS) and nitrogen species, such as polyphenols, flavonoids, terpenoids, and alkaloids (Table 2). These compounds are favored by consumers due to their antioxidant, anti-microbial, anti-inflammatory, anti-viral, anti-parasitic, and immunological properties.

### 3.1. Phenolic Compounds

Gellan gum compounds are a class of important phytochemicals, which are widely present in plants and are believed to have high antioxidant capacity and the ability to scavenge free radicals. CA fruits exhibit high phenolic content and antioxidant capacity, ranking second (405.41 ± 16.70 mg GAE/100 g) for total phenols and fourth for antioxidant activity among 62 fruits [41]. A total of 16 free, 15 bound, and 13 esterified phenolic acids were identified in the CAP using UPLC-ESI-MS/MS detection by Baskaran et al. [26]. CAL features flavonoid glycosides such as rutin and kaempferol/quercetin glycosides [30].

### 3.2. Terpenoids

Terpenoids in CA exhibit strong antibacterial effects, suitable as food preservatives or antibiotics. At least 33 diterpenoids (mostly of the ent-kaurane type) are distributed in peel, pulp, stem, and bark, but are absent in seeds and leaves [42]. Terpenoids from CAL are dominated by sesquiterpenes (21.8% oxygenated and 63.4% hydrocarbons), with γ-cadinene and (E)-caryophyllene as primary constituents [4].

### 3.3. Annonaceous Acetogenins (ACGs)

ACGs are a well-established family of natural products, with many compounds having even stronger anti-tumor activity than paclitaxel, but their toxicity to normal cells prevents clinical use in the treatment of human cancer. Up to now, more than 500 ACGs have been identified in Annonaceae plants (primarily in seeds), such as diepomuricanin A, diepomuricanin B, dieporeticenin, coronin, epoxyrollin A, squamocin P, and annosquatin III, etc. [42]. The ACG extracted from CA has proved to be a potent selective inhibitor of mitochondrial complex I (NADH-ubiquinone oxidoreductase) and is closely related to anticancer activity [4].

### 3.4. Other Bioactive Compounds

Alpha-galactosidase [EC 3.2.1.22] is an exoglycosidase hydrolyzing terminal α-D-galactose from α-D-galactosides, with wide applications in food/beverage industries. Ranganatha et al. (2021) identified an acidic α-galactosidase (CaG, ~67 kDa) from CAS that hydrolyzes α-D-galactosides and binds specific sugars [43]. Pulsed electric field extraction of bioactive compounds from CAL significantly inhibited *Staphylococcus aureus* and *Escherichia coli*, with purpureacin 2 as a key component [35,43].

## 4. Health Benefits

**Figure 2:**
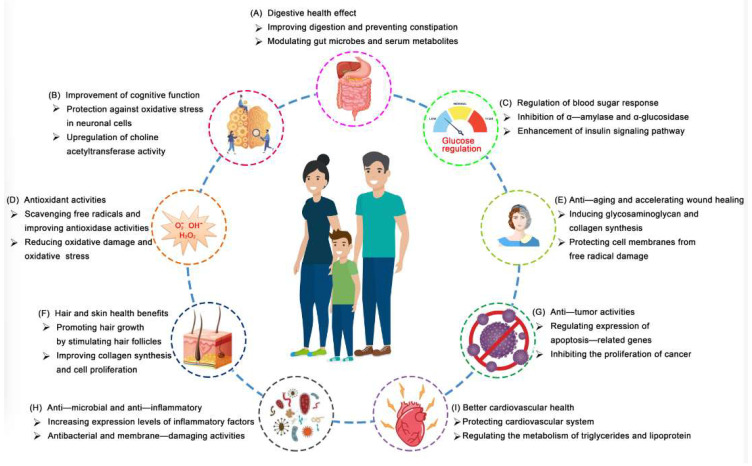
Health benefits of the custard apple and its extracts.

The CA fruit is rich in vitamins, minerals, dietary fiber, and antioxidants that help enhance immune function and promote cardiovascular health. In India, it has been extensively used in folk medicine to treat skin diseases, ulcers, indigestion, arthritis, and tumors, as well as to alleviate pain, repel insects, and combat malaria for a long time [4]. The unripe fruit contains tannins, which are used as astringents in medicines for the treatment of acute diarrhea and dysentery. The crushed leaves were frequently made into a paste for application on boils, blisters, and ulcers. As proven by modern pharmacological experiments, many medicinal properties of the fruit, like anticancer, anti-inflammatory, and regulation of blood sugar response, are primarily attributed to its bioactive compounds, such as flavonoids, terpenes, acetogenins, squamons, and alkaloids [21,44]. In conclusion, CA and its by-product are promising candidates for the development of novel functional foods and drugs, and the health benefits are displayed in Figure 2.

### 4.1. Antioxidant, Anti-Inflammatory, and Wound-Healing Activities

Many diseases are primarily attributed to oxidative stress induced by an imbalance between the formation and neutralization of free radical molecules. Free radicals can directly induce DNA mutations, disruptions in cell signal transduction, cell apoptosis, lipid peroxidation, and degradation of proteins. Natural antioxidants from CA, such as ascorbic acid, tocopherols, flavonoids, and phenols, can help to protect cells from oxidative stress and free them from radical damage [44].

Many studies have shown that substances that have antioxidant properties also have anti-inflammatory effects. Dellai et al. (2010) isolated two cyclic peptides from CAS and confirmed significant anti-inflammatory activity against macrophage J774A by inhibiting levels of IL-6 and TNF-α [45]. In another study, methanol extracts from CAL also showed anti-inflammatory activity by inhibiting IL-6 synthesis in THP-1 cells [18,45]. Numerous studies have confirmed that different parts of CA (bark, leaves, peel, roots, and seeds) demonstrate significant effects in the relief of pain and inflammation, which may be involved in the mechanisms that inhibit the synthesis or release of pro-inflammatory mediators [46]. CAP exhibits pro-healing activity in in vitro assays. In female albino rats, topical application of the CAP extract (100 mg/kg body weight) every other day, for 10 applications over 16 days, significantly accelerated wound healing, which is an effect closely linked to metabolic regulation of hydroxyproline, hexuronic acid, hexosamine, superoxide dismutase, and glutathione in the granulation tissue [47]. In a study of normal and diabetic rats, 200 µL (100 mg/kg body weight) of ethanolic extract of CAL was topically applied on open excision wounds once daily. Wound healing accelerated compared with the control ones during the 16 days of treatment [48]. Excisional wounds were made in Sprague Dawley rats and wound tissues treated with ethyl acetate extract of CAL exhibited enhancement of enzyme activities involved in antioxidant defense, including superoxide dismutase, glutathione peroxidase, and catalase, as well as reduction in malondialdehyde levels of wounds, and notable up-regulation of Hsp70 [49]. In summary, CA is a good source of natural antioxidant substances and can inhibit free radical generation and prevent lipid peroxidation, demonstrating its promising application in the fields of nutraceuticals, dietary supplements, and functional foods.

### 4.2. Anti-Tumor Activities

According to the World Health Organization’s report in 2024, cancer remains humanity’s primary cause of death. In 2022, there were approximately 20 million new cancer cases, and 9.7 million deaths occurred globally. Projections indicate over thirty-five million new cases by 2050, with mortality nearly doubling compared to estimates in 2022 [50]. Plant-derived medicines demonstrate superior anticancer efficacy versus chemotherapy and radiotherapy, which cause severe side effects. CA extracts from pulp, peel, leaf, root, and seed exhibit anticancer activity against multiple human cancer cell lines such as colon, breast, prostate, liver, lung, leukemia, and nasopharyngeal carcinomas. These extracts consistently inhibit cancer cell proliferation and migration while promoting apoptosis through suppressing messenger ribonucleic acid expression (mRNA) of B-cell lymphoma-2 (Bcl-2) and activating the p53/p21CIP1 tumor suppressor pathways [3,18,38,51].

### 4.3. Regulation of Blood Sugar Response

Diabetes mellitus involves chronic hyperglycemia, with herbal foods providing safe therapeutic alternatives. As a traditional Mexican ethnomedicine, CA (pulp, leaves, and peel) has been utilized for adjuvant management of hyperglycemia and diabetes, particularly during early disease stages [52]. It was confirmed that both CAP and CAL extracts exhibit significantly stronger inhibition of α-glucosidase and α-amylase activities compared to the standard drug acarbose [53]. In animal models, peel lyophilizate (100–200 mg/kg) effectively reduced postprandial glucose in healthy Wistar rats and significantly improved non-fasting capillary glucose, glucose intolerance, and insulin response in type two diabetic mellitus rats after a 14-day treatment [54]. Concurrently, leaf hydroalcoholic extract (three hundred fifty milligrams per kilogram) markedly lowered post-load blood glucose levels in both normoglycemic- and alloxan-induced diabetic rats, demonstrating antidiabetic effects comparable or superior to glyburide [55]. These findings collectively substantiate blood-glucose-regulating mechanisms and support its potential as an adjunctive therapy for type two diabetes mellitus.

### 4.4. Improvement of Cognitive Function

CAL shows potential activities for enhancing memory and preventing neurodegenerative disorders. In scopolamine-induced amnesia mouse models, CAL extract modulated the cholinergic system by reducing total acetylcholine and elevating choline acetyltransferase activity, effectively preventing memory impairment linked to oxidative stress and cholinergic dysfunction [56]. In amyloid-β-injected Alzheimer’s disease (AD) models, its ethanol extract significantly suppressed oxidative stress, neuronal death, amyloid-beta aggregation, and memory deficits via the epidermal growth factor receptor and G protein-coupled receptor kinase 2 pathways [57]. Collectively, CAL demonstrates promise as a therapeutic candidate for AD and functional food for cognitive improvement.

### 4.5. Prevention of Cardiovascular and Cerebrovascula Diseases

CA promotes cardiovascular health through multiple mechanisms, and its balanced potassium-to-sodium ratio helps stabilize blood pressure; abundant magnesium enhances myocardial function and relieves cramps, reducing risks of myocardial infarction and stroke. Niacin and dietary fiber regulate cholesterol levels while inhibiting intestinal absorption. Additionally, CA prevents free radical attacks on lipids, supporting heart health [21].

### 4.6. Hair Conditioning

As a traditional remedy for scalp infestation of lice and dandruff in many parts of India, CAS has been used in shampoo or formulated herbal conditioner and has shown positive effects on promoting healthy hair growth, inhibiting scalp inflammation, and preventing hair fall. It might be related to the active compounds (e.g., alkaloids, cyclohexapeptides, acetogenins, etc.) in the seeds [58]. In summary, current research focuses on in vitro experiments, with insufficient verification in terms of animal testing. Most bioactive activities are related to total extracts, and the contribution of single compounds and toxicological thresholds remains to be clarified. In the future, a library of standardized extracts from CA and its by-products should be established, and evaluations of dose–effect–toxicity should be carried out to provide a basis for the product development.

## 5. Industrial Applications

**Figure 3:**
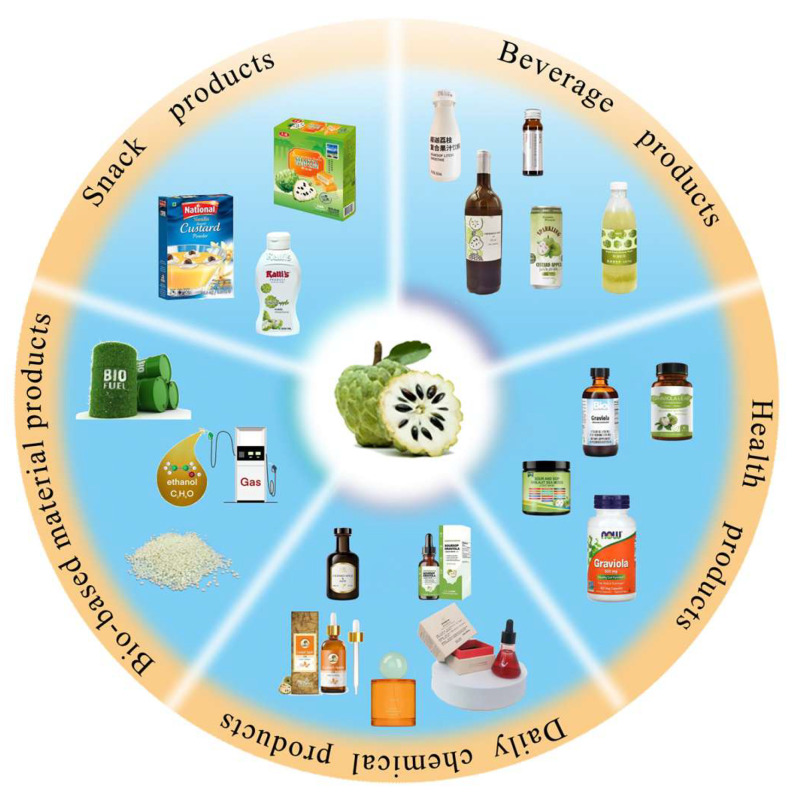
Processed products and potential applications based on custard apple.

The CA fruit is highly perishable (1–2 days of shelf life) due to high respiration rates and inadequate postharvest handling. Browning causes rapid commercial value loss. Cost-effective cold chains are scarce in producing regions, necessitating processing into puree, jam, sweets, liqueur, and ice cream to reduce losses and create market value. Processed products and potential applications based on CA and its by-product are illustrated in Figure 3.

### 5.1. Nutritional Incorporation

In practice, substandard fruits (nutritionally comparable to marketable ones) are always discounted or discarded. Souza et al. (2018) developed CA flour for cookies, showing high acceptance of over 20% daily mineral intake and rich phenolics (200–658 mg gallic acid equivalents per 100 g) [59].

#### 5.1.1. Puree, Jam, and Nectar

Fruit purees enhance flavor and nutrition, especially in foods for infants and toddlers. The global market size was valued at USD 13.9 billion in 2022 and is projected to grow from USD 14.5 billion in 2024 to USD 19.2 billion by 2030. Like many fruits with high sugar content, fresh mature CA was first cleaned, peeled, and deseeded; the pulp was mixed with some necessary additives (e.g., citric acid and nutrients) and then sterilized at 95 °C for 15 min for the puree product. High pressure processing (HPP) inhibits sucrose invertase, pectin methylesterase, and polygalacturonase, yielding a low-glycemic-index puree that delays postprandial glucose peaks in rats [60]. Jam formulations, made up of 60% CA pulp, 40% sugar, and 1.0% citric acid, achieved optimal sensory scores [61]. Nectar blends enrich food nutrition and product diversity [62].

#### 5.1.2. Milkshake

Milkshake (Lassi), a frothy drink of milk blended with flavoring, sometimes fruit, and ice cream, is very popular with children and young people around the world. CAP has been well utilized for the preparation of acceptable milkshake products [63].

#### 5.1.3. Fruit Powder

The production of fruit powder is a practicable solution for fruit processing, which can overcome seasonal unavailability and make the fruit available in different food formulations when desired. The drying techniques, like solar drying, tray drying, spray drying, freeze-drying, and foam-mat drying, can indirectly prolong the storage life of the CA fruit and maintain nutritional and organoleptic properties better [64]. However, quality problems of stickiness, high moisture affinity, and low solubility are still major bottlenecks in the production process due to the presence of high sugar content. In a study, CAP was foamed and dried in a cabinet tray dryer at 50, 60, and 70 °C, respectively, along with 2, 4, and 6 mm of foam thicknesses [65]. The optimum foam expansion (61.11%), foam stability (94%), and lowest foam density (0.67 gm/cm^3^) of CAP were obtained at 3.5% of glycerol monostearate (GMS) and 0.5% of methyl cellulose whipped for 6 min. The tray drying of foamed pulp saves about 180–240 min of drying time compared to the non-foamed one. The formula of 3.5% of GMS and 0.5% of methyl cellulose whipped for 6 min and then dried at 60 °C with a foam thickness of 4 mm was regarded as the perfect treatment for obtaining the pulp powder of CA.

Shrivastava et al. (2021) investigated the effect of process variables on the physicochemical properties of CA powder, and an optimized condition, namely an inlet temperature of 135 °C, an outlet temperature of 75 °C, and a maltodextrin content of 15 g/dL, was obtained and showed the highest powder yield of 14.8%, with the lowest moisture content of 5 g/100 g, as well as a desirable bulk density and solubility index, lesser hygroscopicity, and better stability, which are necessary to ensure a longer shelf life at the commercial level [66]. Additionally, major phytonutrients, namely lutein and zeaxanthin, were retained, which are well-known for their functional properties such as antioxidant, anticancerous, and anti-diabetic activity. Soni et al. (2021) prepared CA powder using convective drying and investigated the physical properties and flow behaviors, obtaining moisture content (7.44%), bulk and tapped densities (359.97 and 423.84 kg/m^3^, respectively), caking degree (55.77%), solubility index (8.00 mL), and Hausner ratio (1.15), and the results showed favorable physical status, appearance quality, and reconstitution effect [67]. CA powder is a promising tablet excipient [67]. One study tested CA powder as an excipient in acetaminophen tablets because of its better flow properties. Compared with standard tablets, which typically use starch as a disintegrator and PVP as a binder, tablets using CA powder and PVP had a 2 times longer disintegration time and a 40% reduction in drug release in the first 15 min [68]. In summary, the development of dietary supplements from CA is critical for providing natural and personalized solutions in the prevention and management of human disease.

#### 5.1.4. Ready to Serve (RTS) Beverages

RTS is a natural beverage for direct consumption and is usually prepared by blending two or more fruit and vegetable juices in different proportions. Some sugar, water, preservatives, or other ingredients are added if necessary. In recent decades, consumers’ demand for RTS beverages, especially personalized ones, has been ever increasing due to changes in lifestyle and dietary habits. In India, CA is always prepared for RTS beverages due to its moderate acidity, sweetness, and high stability; its RTS beverages not only provide a distinct taste and health benefits but also have an extended availability [69].

### 5.2. Fermented Product Applications

Microbial fermentation is a historic key food technology. Probiotic fermentations (e.g., by lactic acid bacteria, LAB) can regulate intestinal microecology and alleviate disease symptoms. CA has been developed into diverse fermented foods.

#### 5.2.1. LAB Fermentation

Tien et al. (2005) demonstrated that LAB-fermented CA juice yields products with unique flavor, appealing texture, high acceptability, and an excellent scavenging capacity in DPPH and iron chelation, indicating functional food potential [70]. Arunkumar et al. (2021) found that CA juice enhances the survivability of microencapsulated probiotics in simulated gastrointestinal tracts without compromising antibacterial activity, acid/bile tolerance, or cholesterol-reducing traits [71]. Thus, LAB-fermented CA juice is an optimal alternative for lactose-intolerant and milk-allergic consumers [71].

#### 5.2.2. Alcoholic Fermentation

CA juice, with high reducing sugars (over 16 Brix), low acidity (pH 4.5), and pleasant sweetness, suits premium fruit wines/brandies/liqueurs. Dilution is usually required due to pulp thickness [72]. Optimized by adding syrup to 24% of total soluble solid (TSS) and 1% of diammonium phosphate (nitrogen source). The beverages fermented by *Saccharomyces cerevisiae* showed appropriate alcohol (5.1–8.14%, *v*/*v*), reduced pH (3.72–5.13), and beneficial compounds like polyphenols (gallic acid and caffeic acid) and vitamin C (up to 45.86 mg/100 mL). Six months of aging significantly enhances sensory qualities (color/aroma/flavor) and antioxidant properties (e.g., DNA radioprotection) [73,74]. Fruit winemaking reduces postharvest losses while adding nutritional and industrial value.

#### 5.2.3. Acetic Fermentation

High sugar content (20%) in CA enables vinegar production via acetic acid bacteria. Raichurkar and Dadagkhair (2017) developed CA vinegar, meeting Indian standards with a pH of 2.8, Brix of 2.0, and acetic acid of (*v*/*v*) 5.39%, with superior sensory acceptance [75]. Such fermentation imparts health benefits (e.g., postbiotic effects) while being eco-friendly and economical [75].

#### 5.2.4. Meat Preservation

CA extracts (peel, seed, leaf) rich in sulfur-polyphenols exhibit excellent antioxidant and antibacterial effects as natural preservatives. Kadam et al. (2018) reported that 0.3–0.5% of CAP extract extended refrigerated chicken breast shelf life from 3 to 9 days (equaling synthetic preservatives) [76]. Leaf extract concentration dependently inhibited pathogens (e.g., *E. coli* and *L. monocytogenes*), delaying microbial growth and lipid oxidation in squid rings and extending shelf life from 6 to 12 days at 4 °C [77]. Despite market potential, further validation in other meats is needed due to the limited literature.

### 5.3. Production of High Added-Value Products

#### 5.3.1. Polysaccharide and Pectin

Polysaccharides from CAP exhibit promising bioactivities. Huang et al. (2023) isolated a new heteropolysaccharide (ASPA80-1) from the pulp; it is mainly composed of glucose and galactose and has a molecular weight of 54.8 kDa [78]. In in vitro tests, it showed immunomodulatory effects like stimulating immune cell proliferation and activation. Overall, polysaccharides demonstrate antioxidant, α-glucosidase inhibitory, and immunostimulant activities, with potential for FSMP. Furthermore, CA is a good natural source of high-quality pectin. Optimized ultrasound-assisted extraction (ultrasonic time 18.04 min, pH 2.36, temperature 63.22 °C, liquid–solid ratio 23.52 mL/g) achieved a yield of 8.93%. The extracted pectin has a high degree of esterification (70–92.3%), high hyaluronic acid content (70.24%), and a molecular weight of 198.65 kDa. Its gel-forming and binding capacities surpass dragon fruit and apple pomace pectin, nearing commercial citrus pectin, making it suitable for snack foods (jam and candies), cosmetics, and many high added-value products [79].

#### 5.3.2. Bio-Based Bioenergy

Biodiesel is an eco-friendly, renewable fossil fuel alternative. Custard apple seed oil (CASO) extracted via consecutive Soxhlet extraction with different solvents can be efficiently transesterified to methyl ester-biodiesel, yielding over 90%. Its performance and emissions meet international standards and are comparable to pure diesel [80,81]. Additionally, CA juice can be fermented to produce bioethanol [82]. Biodiesel production from CA waste is an economical and sustainable method for local clean fuel.

#### 5.3.3. Bio-Based Materials

The by-products of CA, like peels and seeds, are versatile materials. It serves as a biosorbent for removing pollutants (dyes, heavy metals) from wastewater, offering a potential solution for textile industry pollution [83,84]. Seeds also enhance solar evaporation system efficiency [17]. Moreover, CA waste hydrolysate acts as a substrate for fermentative production of biodegradable bioplastics like PHB (yield 7.92 g/L), which degrades completely within 6 weeks [85]. Seed oil can be epoxidized to synthesize plasticized PVC, potentially replacing up to 60% of traditional petroleum-based plasticizers in PVC compounding [86]. Seed oil is also used to synthesize polyurethane coatings via aminolysis with a base catalyst [87]. Utilizing CA by-products for bio-based materials provides a sustainable solution for waste reduction and resource efficiency. Moreover, CAS extraction, as a good capping agent, can reduce the use of dangerous chemicals in the synthesis of the CaO nanoparticle [88].

#### 5.3.4. Biological Regulator

CAS and CAL extracts are effective natural pesticides and growth regulators. Scheunemann et al. (2022) reported that 2000 mg/L of the ethanolic extract of CAS showed ovicidal and larvicidal activities comparable to or better than semi-synthetic insecticides [89]. CAS petroleum ether extract are highly toxic to termites (LC_50_ 2.38–3.06%) and repellent (31.67–48.33%) [90]. Leaf and seed crude oils effectively control maize weevils without harming seed viability [91]. Additionally, CAL extract (3%) as a feed additive can improve rooster performance and suppress harmful gut bacteria [92]. These bio-based extracts are environmentally friendly options for integrated pest management; future studies should explore broader crop applications.

## 6. Potential for Toxicity

Although the CA fruit is edible, some parts of the CA tree may be toxic. For instance, the leaves, seeds, and young fruits have been used as insecticides for some pests. If eyes are accidentally exposed to sap, it can cause irritation and even corneal abrasions. It was proven that the extraction of CAS contains alkaloids and other toxins, which can easily reach the flesh and cause poisoning when the seeds are damaged. CAL showed no genetic toxicity in both in vitro and in vivo toxicity assessments; however, it could cause genetic mutations in some *Salmonella typhimurium* strains. Sohn et al. [93] speculated that the genotoxic effects of CAL are related to cellular keratinization, nucleotide metabolism, and electron transport chain activities. In acute and subacute toxicity assays, they found that the oral median lethal dose (LD_50_) of CAL extract is higher than 2000 mg/kg per day in male and female rats [94]. In a two-year experiment, a custard apple seed powder (CASP)-mixed diet was fed to mature Rattus at 40 g/day at 10 weeks of age, and a sterility test was carried out on 38- and 28-week-old rats in the first and second years. No pregnancy or parturition occurred when they were fed with a normal diet without CASP during the whole experimental period, and CASP was considered to have the potential of reducing the rodent populations [95].

In the past, CAS was used for the manufacturing of lubricant and soap, which has been a traditional remedy for scalp infestation of lice and dandruff in some parts of South India for many years. During this process, the CASP is quite possibly inducing toxic keratitis due to ocular exposure [96,97]. In six case reports, four patients had used CASP to treat head lice and hair loss, and one patient had utilized CASP to prevent acne, and all of them exhibited symptoms of toxic keratitis, including eye pain, watering, and photophobia, concluding that CAS is highly toxic to ocular structures, especially the conjunctiva and corneal epithelium. Despite the bioactive compounds from CA and its by-product showing various medicinal effects, inappropriate exposure can sometimes cause severe toxicity with life-threatening consequences, and scientific use in folk medicine and public awareness of the adverse effects are needed.

## 7. Conclusions and Future Perspectives

Commercial CA products (e.g., desserts, purees, and beverages) are increasingly popular, with novel processing technologies and optimized distribution enhancing consumer satisfaction and market vitality. The survey of the literature based on the phytochemical profile and health benefits of CA showed that it can also be used as potential ingredients in functional foods and pharmaceutical drugs. Nevertheless, critical research gaps persist. Comprehensive studies on postharvest quality encompassing chemical profiles and bioactive properties of all fruit components (pulp, peel, seed, and leaf) are essential to advance health-focused food innovation. Systematic collection of varietal nutritional and processing data is equally crucial to drive product development and integrate industrial chains. (A) Varietal development and database construction. Varietal characteristics dictate raw material quality and processing suitability. Current data on physicochemical properties across CA varieties and origins remain insufficient. Establishing a dedicated database through targeted research is imperative. Most cultivars are consumed fresh, and dedicated processing varieties are still absent. Artificial intelligence-assisted breeding offers a strategic pathway to accelerate the development of processing-optimized cultivars. For example, Thite et al. (2025) have established a comprehensive CA disease dataset for precise detection by using refined machine learning algorithms such as deep learning, feature extraction, and pattern recognition [98]. This dataset fosters collaborative efforts, aiding disease prevention techniques to boost custard apple yield and refine farming [98]. (B) Processing technology and equipment. Developing cost-effective specialized equipment and scalable processing systems is critical. Tropical/subtropical regions frequently lack adequate processing infrastructure. Modern techniques (extraction, enzymolysis, fermentation, freeze-drying, etc.) face adoption barriers due to high costs and operational complexity, particularly for smallholders. Innovating affordable, adaptable technologies is fundamental to unlocking the industrial potential of CA. (C) Transformation mechanisms during processing. The rich composition (carbohydrates, proteins, organic acids, and polyphenols) of CA necessitates deeper investigation into processing-induced nutrient transformations. Key unknowns include how flavor dynamics and how interactions among bioactive compounds affect product stability and functionality. Multi-omics methodologies can elucidate quality evolution mechanisms and predict nutritional, functional, and sensory changes. Integrated tools like FLAVOUR-AI may de-risk process optimization and enable precision manufacturing of value-added products. (D) Pharmacological mechanisms. As a folk medicinal plant in the tropics, CA has long been used to treat many ailments. However, the exact mechanism of action between conventional medicine and treatment is still unclear. The dosage–effect relation and biological mechanism in therapeutics are still not fully understood. Systems pharmacology and molecular docking represent promising approaches to resolve these uncertainties, clarify holistic mechanisms, and guide rational clinical utilization. (E) Industrialization challenges. While CA extracts show bioactivity in vitro and in vivo tests, barriers impede its commercialization due to the absence of rigorous clinical efficacy and safety validation. Future studies will focus on natural products with therapeutic potential to formulate such as FSMP, personal care, and cosmetics.

## Figures and Tables

**Table 1:** Nutrient compositions in different parts of custard apple.

Classifications	Nutrition Compositions	Methods of Detection	Pulp	Seed	Leaf	References
			Contents (Dried Weight)			
Carbohydrate	Total carbohydrate	AOCS official method	18.2–59.0 g/100 g			[8,9,10]
	Crude fiber		2.55–11.0 g/100 g	16.80 g/100 g		
Amino acids	Threonine	Amino acid analyzer		5.67 g/100 g		[11]
	Valine			11.24 g/100 g		
	Isoleucine			8.12 g/100 g		
	Leucine			14.79 g/100 g		
	Histidine			2.43 g/100 g		
	Lysine			7.12 g/100 g		
	Arginine			12.32 g/100 g		
	Glutamic acid			17.41 g/100 g		
	Serine			5.23 g/100 g		
	Glycine			6.86 g/100 g		
	Aspartic acid			11.97 g/100 g		
	Alanine			6.86 g/100 g		
Fatty acids	Palmitic acid (C16:0)	GC-FID, GC-MS		1.82–4.65 g/100 g		[11,12,13]
	Stearic acid (C18:0)			1.12–2.76 g/100 g		
	Oleic acid (C18:1)			7.12–13.13 g/100 g		
	Linoleic acid (C18:2)			3.45–7.57 g/100 g		
Vitamins	Ascorbic acid	HPLC-MS/MS	9.22–300 mg/100 g		0.01–0.02 mg/g FW	[4,8,9,10,11,14,15,16,17,18]
	Thiamine		0.05–0.28 mg/100 g			
	Riboflavin		0.07–0.28 mg/100 g			
	Niacin		0.80–2.2 mg/100 g			
	Panthothenic acid		0.20–1.28 mg/100 g			
	Pyridoxine		0–0.5 mg/100 g			
	Folic acid		35.00 µg/100 g		8.12–11.98 g/g FW	
	Vitamin B12		0.057–0.167 mg/100 g			
	Vitamin A		1.80–7.00 µg/100 g			
	Vitamin E		0.60 mg/100 g	15.50–16.60 mg/100 g		
Minerals	Ca	FAES, ICP-MS/MS	4.92–75.47 mg/100 g	3.41–68.79 mg/100 g	0.28 g/100 g	
	P		20.00–235.61 mg/100 g	328 mg/100 g		
	Fe		0.30–105.00 mg/100 g	1.09 mg/100 g	37.23–49.55 µmol/g	
	K		250–1362.25 mg/100 g	252.47–386.98 µmol/g	363 mg/100 g	
	Na		4.50–62.75 mg/100 g	61 mg/100 g	61.2–95.18 µmol/g	
	Mg		21.0–64.45 mg/100 g	98 mg/100 g		
	Cu		0.11–2.75 mg/100 g	1.09 mg/100 g		
	Mn		0.21–1.75 mg/100 g	2.93 mg/100 g		
	Zn		0.57–1.21 mg/100 g	2.84 mg/100 g		
	Ba		0.028–0.23 mg/100 g			
	Se		1.50 µg/100 g			

**Table 2:** Key bioactive compounds in different parts of the custard apple and biological activities.

Parts	Compounds	Biological Activities Identified	Type of Study	References
Pulp	**Ent-kaurane diterpenes** (e.g., 16β,17-dihydroxy-ent-kauran-19-oic acid, 17-hydroxy-16β-ent-kauran-19-al)	Anti-HIV, anti-tumor	In vivo against 95-D lung and ovarian A2780 cancer cells	[24,25]
	**Phenolics and derivatives** (e.g., gallic acid, ferulic acid, protocatechuic acid, caffeic acid, p-coumaric acid, sinapic acid, quinic acid, decycloxybenzoic acid, procyanidin B2, procyanidin trimer, catechin, epicatechin, epigallocatechin gallate, 4-(β-D-glucopyranosyloxy) benzoic acid, epigallocatechin, 7-hydroxycoumarin 7-glucoside, dihydroquercetin, xanthotaxol acetate, caffeoyl hexoside)	Antioxidant	In vitro, rats and mice	[26]
Peel	**Fatty acids and diterpenes** (e.g., (9Z)-9-octadecenoic acid, ent-kaur-16-en-19-oic acid, (-)-ent-kaur-16-en-19-oic acid, 16α,17-dihydroxy-ent-kauran-19-oic acid, 4β,17-dihydroxy-16α-acetoxy-18-nor-ent-kaurane)	Anti-tumor	In vivo against SMMC-7721 and HepG2 cell line	[27,28,29]
	**Azulene derivative (e.g., 1H-cycloprop[e]azulen-7-ol** decahydro-1,1,7-trimethyl-4-methylene-[1ar-(1aα, 4aα, 7β, 7a, β, 7bα)])	Anti-parasitic activity	In vitro	[24]
Leaf	**Flavonoids and derivatives** (e.g., rutin kaempferol-3-O-rutinoside, quercetin-3-O-robinobioside, quercetin-3-O-β-D-glucoside, quercetin-3-O-glucoside)	Antioxidant and hypoglycemic activity	In vitro, diabetic rats	[30,31]
	**Acetogenins** (e.g., annoreticuin, isoannoreticuin)	Anti-tumor	In vivo against AD-5 tumor	[32]
	**Flavonoid derivatives** (e.g., 5,7,4′-trihydroxy-6,3′dimethoxy-flavone 5-O-α-L-rhamnopyranoside (THDMF-Rha)	Hepatoprotective	Cellular levels, rats	[33]
	**Acetogenins** (e.g., annotemoyin, purpureacin 2	Anti-microbes	In vivo against H22 liver cancer mice	[34,35]
Seed	**Diterpenes** (e.g., 16-ahydroxy-(−)-kauran-19-oic)	Anti-microbes	In vitro	[36]
	**Cyclopeptides** (e.g., cyclosquamosin D)	Anti-inflammatory	In vitro	[37]
	**Acetogenins** (e.g., dieporeticenin B, squamocin P, annosquatin III, annonacin)	Anti-tumor (cytotoxic)	In vitro cytotoxic activity, cellular levels	[38,39]
	**Phenolic and Flavonoids** (e.g., quercetin, ferulic acid, kaempferol, 6-methoxy isovitexin)	Antioxidant	In vitro	[40]

## Data Availability

No new data were created or analyzed in this study.

## Supplementary Materials

The following supporting information can be downloaded at: https://www.mdpi.com/article/10.3390/foods14193413/s1, Figure S1. PRlSMA flow diagram of the literature search and selection process.
