# Supplementary figures and images for: A Review of Nutrition, Bioactivities, and Health Benefits of Custard Apple (Annona squamosa): From Phytochemicals to Potential Application
Source: Foods. 2025 Oct 2;14(19):3413. doi: 10.3390/foods14193413 (PMC12523297; doi:10.3390/foods14193413)

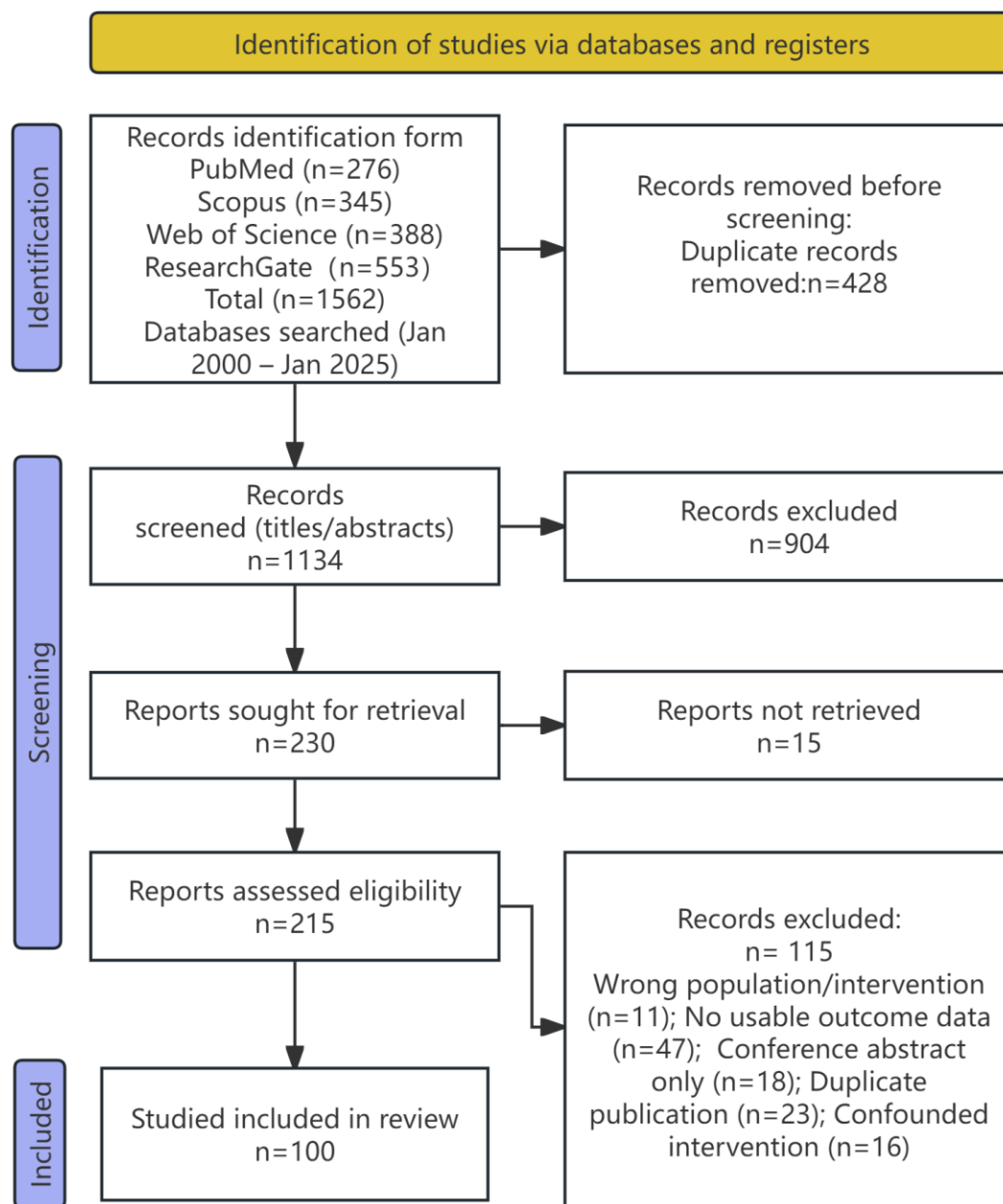

Figure S1. PRISMA flow diagram of the literature search and selection process.

Supplement: Supplementary file 1 [file foods-14-03413-s001.zip › foods-3850456-supplementary.pdf]
